# Compartmentalization in PVC super-phylum: evolution and impact

**DOI:** 10.1186/s13062-016-0144-3

**Published:** 2016-08-09

**Authors:** Sandrine Pinos, Pierre Pontarotti, Didier Raoult, Jean Pierre Baudoin, Isabelle Pagnier

**Affiliations:** 1 Aix Marseille Université, URMITE, UM63, CNRS 7278, IRD 198, INSERM 1095, 27 Bd Jean Moulin, 13385 Marseille Cedex 5, France; 2 Aix Marseille Université, CNRS, Centrale Marseille, I2M UMR 7373, Evolution Biologique et Modélisation, 13385 Marseille, Cedex 5, France

**Keywords:** Evolution, Bacteria, PVC-super phylum, Microscopy, Compartmentalization

## Abstract

**Background:**

The PVC super-phylum gathers bacteria from seven phyla (*Planctomycetes, Verrucomicrobiae, Chlamydiae, Lentisphaera, Poribacteria, OP3, WWE2*) presenting different lifestyles, cell plans and environments. *Planctomyces* and several *Verrucomicrobiae* exhibit a complex cell plan, with an intracytoplasmic membrane inducing the compartmentalization of the cytoplasm into two regions (pirellulosome and paryphoplasm). The evolution and function of this cell plan is still subject to debate. In this work, we hypothesized that it could play a role in protection of the bacterial DNA, especially against Horizontal Genes Transfers (HGT). Therefore, 64 bacterial genomes belonging to seven different phyla (whose four PVC phyla) were studied. We reconstructed the evolution of the cell plan as precisely as possible, thanks to information obtained by bibliographic study and electronic microscopy. We used a strategy based on comparative phylogenomic in order to determine the part occupied by the horizontal transfers for each studied genomes.

**Results:**

Our results show that the bacteria *Simkania negevensis* (*Chlamydiae*) and *Coraliomargarita akajimensis (Verrucomicrobiae*), whose cell plan were unknown before, are compartmentalized, as we can see on the micrographies. This is one of the first indication of the presence of an intracytoplasmic membrane in a *Chlamydiae*. The proportion of HGT does not seems to be related to the cell plan of bacteria, suggesting that compartmentalization does not induce a protection of bacterial DNA against HGT. Conversely, lifestyle of bacteria seems to impact the ability of bacteria to exchange genes.

**Conclusions:**

Our study allows a best reconstruction of the evolution of intracytoplasmic membrane, but this structure seems to have no impact on HGT occurrences.

**Reviewers:**

This article was reviewed by Mircea Podar and Olivier Tenaillon.

**Electronic supplementary material:**

The online version of this article (doi:10.1186/s13062-016-0144-3) contains supplementary material, which is available to authorized users.

## Background

The PVC super-phylum gathers seven bacterial phyla *(Planctomycetes, Verrucomicrobiae, Chlamydiae, Lentisphaerae, Poribacteria, OP3, WWE2*) [1–4], and comports 37 species of bacteria whose genome was entirely sequenced. The monophyly of this super phylum have been discussed a lot in the last years, due to the difficulty to obtain a consensual phylogeny [5–14]. Recently, it seems that a global consensus was reached, that include all these bacteria in a same super-phylum [2, 5]. This idea is confirmed by the discovery in 2012 and 2014 of a molecular signature conserved in all PVC bacteria [1, 15]. The phylogenetic relations between PVC and other bacteria are still a controversial subject [5, 13]. PVC bacteria are present in an important variety of environments: water, soils, vertebrates, amoeba, insects… [5, 16–21].

The bacteria of this super-phylum show different interesting characteristics [11, 22]. Some of these features can be found in other bacteria: some *Planctomycetes* are implicated in carbon and nitrogen cycles [23, 24] or synthesize special sterols [25], many *Chlamydiae* are pathogens of mammals [26, 27], some *Chlamydiae* and *Verrucomicrobiae* are symbionts [19]. But PVC present also genetic and cellular features unusual among bacteria: compartmentalization in *Verrucomicrobiae* and *Planctomycetes* [28, 29], absence of tubulin like protein FtZ in *Planctomycetes* and *Chlamydiae* [30, 31], crateiform surface and budding reproduction [32] in *Planctomycetes*. Among these characteristics, we were specifically interested in the compartmentalization of bacteria. This feature is characterized by a specific cell plan, and concerns all the *Planctomycetes* [29, 33, 34], some *Verrucomicrobiae* [28], one *Lentisphaera* and one *Poribacteria* [35]. An intracytoplasmic membrane (ICM) separates the cytoplasm of bacteria into two distinct compartments: the pirellulosome inside (containing the DNA [36]), and the paryphoplasm outside (the size and shape of these two compartments varied a lot among PVC bacteria). This ICM is a lipid bilayer in contact with proteins [29, 32, 33] presenting structural similarities with some proteins from eukaryotic membranes, such as clathrins [37, 38]. Some compartmentalized bacteria also present a more complex cell plan, with the presence of another compartment, like the anamoxosome in *Candidatus Kuenenia Stuttgartiensis* [32, 39, 40], or a double internal membrane with ribosomes [41] in the same compartment that DNA [36] in *Gemmata obscuriglobus* [42]. Compartmentalization in PVC bacteria is still debatable and three opinions are defended actually : 1- As some features of PVC bacteria are current in *Eukaryota* [15], this observation allows some people to assume that the compartmentalization of PVC is the precursor of Eukaryotic nuclei, but this idea is not very popular [34, 43–48]. 2- Another proposition is that the compartment is structurally and functionally similar to a nucleus but that these two structures appeared independently [49]. 3- Some people identify the membrane as an invagination of a Gram negative external membrane [50].

Considering the reality of the existence of a compartmentalization in PVC bacteria, it would be interesting to determine the function of this specific cell plan; indeed, no previous studies had been able to determine it. Here we assumed that this membrane could be a protection against Horizontal Gene Transfers (HGT). This hypothesis was based on observations of the impact of different membranes already studied : the role of the internal membrane in Eukaryotes, or the influence of host membrane on genomes from intracellular bacteria (which limits the contact between foreign elements and DNA). We used a phylogenomic strategy of HGT detection to reconstruct the history of HGT during the evolution of PVC bacteria, before and after appearance and disappearance of compartmentalization.

## Results

### Lifestyle of species and evolution of cell plan

PVC bacteria present two different cell plan, compartmentalized by an intra cytoplasmic membrane (ICM) or non compartmentalized. They also present two different lifestyles: allopatric lifestyle, for bacteria isolated from other microorganisms (obligate intracellular bacteria living in non amoeba cells) and sympatric, for bacteria in contact with other microorganisms. The studies already published allowed determining cell plan of 26 PVC bacteria: PVC super phylum presented 18 compartmentalized species distributed in three phyla (*Verrucomicrobiae, Planctomycetes* and *Lentisphaerae*) and eight non compartmentalized bacteria in one phyla (*Chlamydiae*). These bacteria were used in our study, but we also selected seven bacteria with an unknown cell plan, distributed in two phyla (*Verrucomicrobiae* and *Chlamydiae*). These data allowed the reconstruction of cell plan evolution with a good reliability, however it was still difficult to conclude about ancestral state at different nodes of the tree, especially in *Chlamydiae* phylum (their common ancestor presented a probability of 80.2 % to be compartmentalized). It was interesting to add new information in the dataset, in order to improve this reconstruction.

**Fig. 1:**
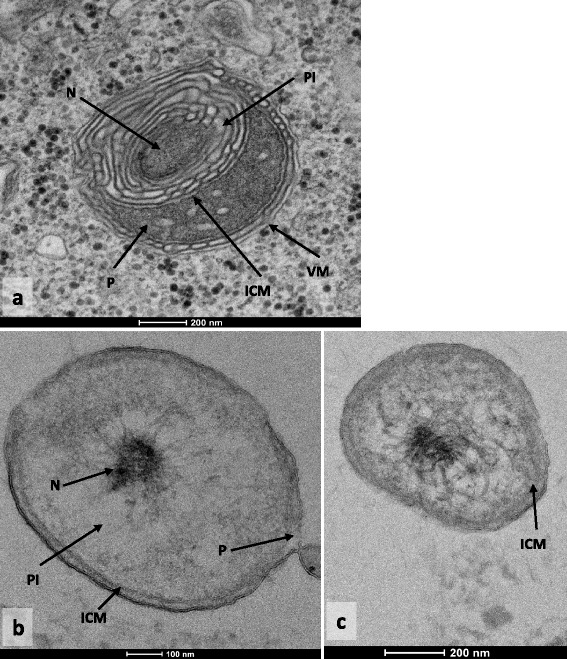
**a** Transmission electron micrograph of thin section of cell of Simkania negevensis. Pirellulosome (PI) and paryphoplasm (P) are separated by the intracytoplasmic membrane (ICM). The nucleoid (N) is contained within the pirellulosome. The bacterium lives in the cytoplasm of an amoeba and is surrounded by the membrane of phagocytic vacuole (VM). Bar marker, 0.2 μm. **b**) **c**): Transmission electron micrograph of thin section of cell of *Coraliomargarite akajimensis*. Pirellulosome (PI) and paryphoplasm (P) are separated by the intracytoplasmic membrane (ICM). The nucleoid (N) is contained within the pirellulosome. Bar marker, 0.2 μm

The electron microscopic pictures obtained for the thin sections of *Simkania negevensis* (Fig. 1a) and *Coraliomargarita akajimensis* (Fig. 1b, c), revealed the presence of a potential intracytoplasmic membrane in these two bacteria. In both cases, cells contain structures identified as a pirellulosome and a paryphoplasm, separated by the intracytoplasmic membrane. The nucleoid is contained within the pirellulosome. For *S.negevensis*, we noticed some differences compared to the other compartmentalized species: we identified the membrane of the phagocytic vacuole surrounding the *Chlamydiae*, related to the phagocytosis of *S.negevensis* by the amoeba. An important presence of internal membranes is also detected. *C.akajimensis* presents a compartmentalized cell plan with an intracytoplasmic membrane close to the external membrane.

**Fig. 2:**
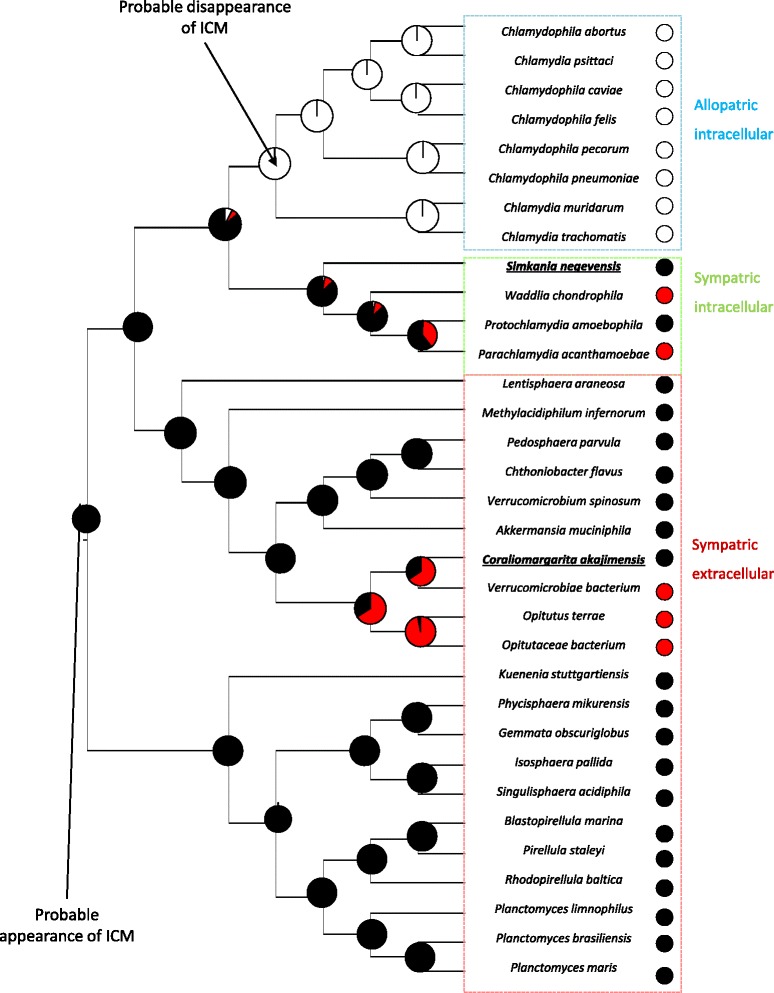
Phylogeny of PVC super-phylum with indications of cell plan and lifestyles for studied species. The points at the nodes indicate the cell plan: *black* for compartmentalization, *white* for no compartmentalization and *red* for unknown cell plan. The color squares indicate the lifestyles of bacteria. The probabilities of the different cell plan states in ancestors are presented by diagrams, the portions of the different colors indicates their probabilities

These microscopic observations revealed cell plan information about two bacteria, whose a *Chlamydiae*. allowing us to better reconstruct the ancestral state of cell plan all along the PVC super-phylum evolution (Fig. 2). The compartmentalization of *S.negevensis* induces an increase in the probability of compartmentalization in the *Chlamydiae* last common ancestor to 86.1 % (against 80.2 % without this information). The ancestral reconstruction permitted to date the appearance of intracytoplasmic membrane to the root of PVC super-phylum with a probability of 99.8 % (against 96.1 % without our new data). The eight non compartmentalized *Chlamydia*e form a monophyletic group and represents all the allopatric bacteria of the super-phylum, their ancestor was supposed to be allopatric and was predicted as non compartmentalized, suggesting a disappearance of compartmentalization in this ancestor (with a probability of 99.4 %).

### Relation between cell plan and genomes contents

**Fig. 3:**
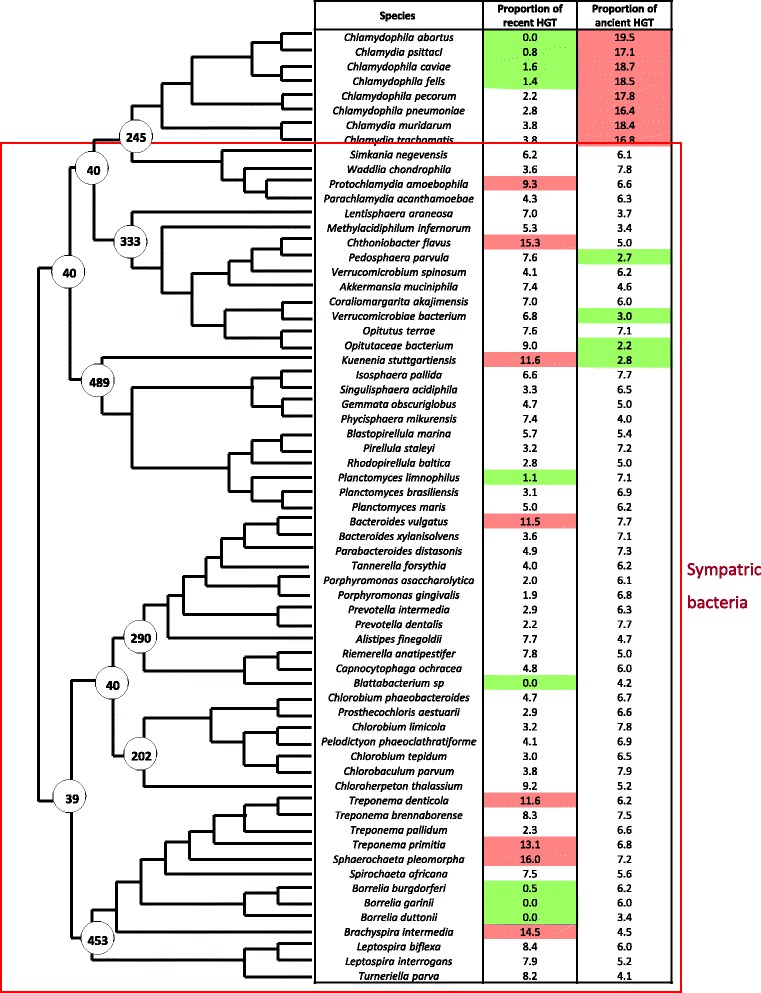
Proportion of genes acquired by transfer in the genomes of the 64 bacteria studied. This figure presents the proportion of HGT that occurred recently and formerly, in each genome of studied bacteria. The points at the nodes of phylogeny show the quantity of HGT occurred between the node and the following. The rates of recent and ancient HGT are presented in tables, a high rate of HGT is indicates by a *red* color, a low rate by *green*

HGT were detected by phylogenomic methods, the position of HGT in species tree allows to differentiate HGT occurred only in modern species (specific HGT) and HGT occurred in their ancestors (non specific HGT). We identified 27275.0 proteins acquired by HGT (13.2 % of all proteins in proteomes studied). The proteomes of sympatric compartmentalized bacteria present 13040.0 HGT with 7259.0 specific HGT (6.2 % of proteomes) and 5781.0 non specific (4.9 % of proteomes). The proteomes of sympatric non compartmentalized bacteria contain 12686.0 HGT, whom 5466.0 specific (6.7 % of proteomes) and 7220.0 non specific (8.9 % of proteomes). Allopatric non compartmentalized bacteria present 1549 proteins acquired by HGT in their proteomes, with 160 specific HGT (2.0 % of proteomes) and 1389 non specific HGT (17.7 %). When we compared their HGT proportions (Fig. 3), we detected a significantly lower proportions of specific HGT in allopatric bacteria, compared to sympatric bacteria (ANOVA test: *p*-value = 3.0*10-4). In species tree, an important decrease of HGT proportions was identified after the conversion of *Chlamydiae* to intracellular allopatric lifestyle. Among sympatric bacteria, we could not identify a significant difference of HGT proportion according cell plan, regardless the type of HGT studied (total / specific / non specific HGT; Kruskal test or ANOVA test, *p*-value = 3.1*10-1 / 9.8*10-1 / 5.1*10-1 respectively) (Fig. 3). The quantities of HGT were compared between the ancestors of studied bacteria, according to their most probable cell plan. This comparison did not allow to detect a significant differences between ancestral bacteria depending on cell plan (ANOVA test, *p*-value = 6.2*10-1). These observations suggest that intracytoplasmic membrane has no impact on HGT frequency.

The compartmentalization of bacteria could disturb only some particular mechanisms of HGT processes, leading to a low impact on HGT global proportions. The study of mobilome revealed that no element present proportions varying significantly according cell plan (Additional file 1). The lifestyle of bacteria seems to clearly influence the variations of some mobilome elements; indeed, the intracellular bacteria present more abundant proportions of transposases than extracellular bacteria (Kruskal test : *p*-value = 2.0*10-2). Conjugation genes are significantly over represented in the genomes of Intracellular allopatric bacteria, and underrepresented in extracellular compartmentalized bacteria (Kruskal test : *p*-value = 3.0*10-2).

## Discussion

### Lifestyle of species and evolution of cell plan

The results of electronic microscopy allowed to defined *S.negevensis* and *C.akajimensis* as compartmentalized bacteria. The *Chlamydiae* is characterized by an important presence of internal membranes. This observation is probably related to the folds of the intracytoplasmic membrane, as it is visible for the *G.obscuriglobus* in the micrographies and the three dimensional reconstruction published by Santarella-Mellwig et al in 2010 [38] and 2013 [37] respectively. Conversely, the cell plan observed is different from that identified in the cryotomographies realized by Pilhofer et al in 2014 [51]. The differences could be related to the variations in methodology used and in the growth stage observed. The possible compartmentalization of *S.negevensis* is very interesting because the cell plan is unknown in a large majority of sympatric *Chlamydiae. C.akajimensis* presents a more classical compartmentalized cell plan, as it was already observed in some PVC bacteria by Santarella-Mellwig in 2013 [37] and Kuo-Chang Lee and al in 2009 [28]. These micrographies allowed an improving in reconstruction of cell plan evolution among PVC bacteria, especially in *Chlamydiae*. They permitted to analyze the impact of ICM in the phyla of intracellular sympatric *Chlamydiae*, with two bacteria presenting a known cell plan and the probable presence of compartmentalization in their last common ancestor (with a probability of 71 %, against 57 % without the information about *S.negevensis*). The micrography of *C.akajimensis* completes the studies already realized on the *Verrucomicrobiae* and reinforces the probability of compartmentalization in the last common ancestor of *Verrucomicrobiae* (probability of compartmentalization = 83 %, against 79 % without the cell plan of *C.akajimensis*). These observations allowed to conclude with fewer uncertainties our study concerning the HGT in compartmentalized bacteria.

Cell plan evolution reconstruction indicated that compartmentalization appeared at the root of PVC super-phylum and disappeared once, in the group of allopatric intracellular bacteria. This disappearance seems to occurred simultaneously with bacteria conversion to intracellular allopatric lifestyle. Intracellular allopatric bacteria are isolated from other bacteria, so their genomes are protected against HGT. Conversely, extracellular and intracellular sympatric bacteria are more exposed to exchanges because they are in contact with many microorganisms. An hypothesis could be that the intracytoplasmic membrane plays a role in protection of genomes against HGT, as host membrane of intracellular bacteria or eukaryotic membrane [52]. This intracytoplasmic membrane became useless in allopatric bacteria, due to the absence of HGT, leading to its disappearance. However, this hypothesis needs to be confirmed by data concerning genomes evolution in compartmentalized and non compartmentalized bacteria.

### Relation between cell plan and genomes contents

The analysis of HGT in the different groups of bacteria highlighted the role of lifestyle on HGT process and the absence of impact of compartmentalization. The important decrease of HGT proportions observed in *Chlamydiae* after their conversion to the intracellular allopatric lifestyle is probably due to the physical isolation from other microorganisms, which prevents the opportunities of HGT [52]. This agrees with previous studies showing that the predominant evolutionary process in intracellular bacteria is genome reduction leading to small genome sizes [53, 54] with the bacteria from amoeba as the exception [55, 56]. The similarity of HGT proportions between compartmentalized and non compartmentalized bacteria suggests that the presence on an intracytoplasmic membrane has no impact on HGT process. We could imagine that this absence of difference is due to a problem of our strategy but three observations demonstrate the efficiency of our method: *Chlamydiae* present an important rate of ancient HGT with eukaryotes, significantly higher than other bacteria (ANOVA test *p*-value = 3.3 × 10-6). This observation is consistent with the existing literature dedicated to transfers between plants and *Chlamydiae* [57, 58] that supports a role of *Chlamydiae* in chloroplast endosymbiosis; The HGT proportions detected in two *Planctomycetes* (*Planctomyces maris* and *Candidatus Kuenenia stuttgartiensis*) correspond to these observed by Kamneva et al in 2012 [5]; *Spirochaetes* seem to exchanges more with *Firmicutes* as it was already detected in previous studies [59, 60].

A similar HGT proportion in compartmentalized and non-compartmentalized bacteria does not necessary contradict the hypothesis of protection against HGT by compartmentalization. Indeed, the intracytoplasmic membrane of PVC bacteria can represents a barrier to HGT by disturbing only some transfers mechanisms mediated by mobilome elements. This perturbation could lead or not to a decrease of HGT level, depending if the deficiency of some mechanisms are offset by the others. The absence of difference in mobilome elements quantities and proportions between the different groups of bacteria indicated that the compartmentalization has no impact, not only on HGT proportions, but also on elements implicated in HGT process. The lifestyle seems to have an influence on two mobilomes elements, proportions of transposases and conjugation genes. The high proportion of transposases in intracellular allopatric bacteria could be related to the importance of transposable elements in genomes of intracellular bacteria [61, 62]. However, conjugation genes present other uses in bacteria than conjugation [63, 64]. So we can assume that the abundance of conjugation genes in bacteria has probably no influence on the HGT.

The function of compartmentalization in PVC bacteria remains unknown and the causes of intracytoplasmic membrane disappearance in intracellular isolated bacteria is still unresolved.

## Conclusions

The different pictures obtained for *Verrucomicrobiae* and *Chlamydiae* allow a best definition of ancestral and modern states of compartmentalization and so, permit to reconstruct intracytoplasmic membrane evolution in PVC bacteria. But this compartmentalization seems have no significant impact on HGT quantity, HGT proportion or partners of transfers.

## Methods

### Bacteria selection and genomes recovery

We selected bacteria from 4 phyla of PVC super-phylum: *Planctomycetes, Verrucomicrobia, Lentisphaerae*, and *Chlamydiae.* Bacteria from three phylogenetically close phyla were chosen as negative controls, based on a reference tree [5]: *Bacteroidetes, Spirochaetes* and *Chlorobi*. We retrieved the proteomes of all bacteria selected in genomes NCBI database (Additional file 2).

### Lifestyle and cell plan determination

The lifestyle of selected bacteria is determined by a bibliographic study of each bacterium. Two types of lifestyles are known for bacteria, according the possibility of exchanges with other microorganisms: allopatric or sympatric bacteria [65, 66]. Sympatric bacteria are bacteria living in community with other microorganisms, they exchange easily genes by lateral transfer through their interactions. Allopatric bacteria are living in cells and are not in contact with other microorganisms, more specialized, with a reduced genome size and less genetic exchanges. They are obligate intracellular bacteria, living in non amoeba cells.

Cell plans of bacteria are determined thanks to transmission electron microscopy pictures already available in bibliography [28, 29, 34, 35] and microscopic observations of the bacteria whose cell plan is unknown. First, the species *Simkania negevensis* (DSM27360^T^, type strain) was grown within a culture of its amoebal host *A. castellanii.* At H6, and H16 post-infection, cultures were centrifuged at 2000 rpm/min during 10 min, and the pellets were fixed for electron microscopy in a fixative solution (2.5 % glutaraldehyde in 0.1 M sodium cacodylate buffer). The second species, *Coraliomargarita akajimensis* (DSM 45221^T^, type strain) was cultivated directly into Bacto Marine Broth medium (Difco). Growth occurred after 6 days of cultivation, and when the bacterial suspension reached the exponential growth phase, it was centrifuged at 5500 rpm/min during 30 min, and fixed for electron microscopy in the same fixative solution. An aliquot of each culture was kept for DNA extraction, followed by a standard 16S rRNA PCR and sequencing, in order to confirm the bacterial identification. For both bacteria, Electron microscopic observations were realized in different steps: for embedding, cells were fixed for 1 h with glutaraldehyde 2.5 % in 0.1 M sodium cacodylate buffer and washed three times. Cells were post-fixed for 1 h with 1 % OsO4 diluted in 0.2 M Potassium hexa-cyanoferrate (III) / 0.1 M sodium cacodylate solution. After four 5 min washes with distilled water, cells were gradually dehydrated with ethanol by successive 10 min baths in 30, 50, 70, 96, 100 and 100 % ethanol. Substitution was achieved by successively placing the cells in 25, 50 and 75 % Epon solutions for 15 min. Cells were placed for 1 h in 100 % Epon solution and in fresh Epon 100 % over-night under vacuum at room-temperature. Polymerization took place with cells in fresh 100 % Epon for 24 h at 60°. Ultrathin 70 nm sections were cut with a UC7 ultramicrotome (Leica) and placed on HR25 300 Mesh Copper/Rhodium grids (TAAB, UK). Electron micrographs were obtained on a Tecnai G20 TEM operated at 200 keV equipped with a 4096 × 4096 pixels resolution Eagle camera (FEI)).

### Phylogeny and cell plan evolution reconstruction

We reconstructed the species tree of PVC bacteria and *Bacteroidetes-Chlorobi-Spirochaetes* thanks to 12 markers common to the 65 species (three ribosomal proteins (16S, 23S, 30S), two elongation factors (Tu and Ts), two DNA polymerase subunits, CTP synthetase, and four tRNA ligases). The selected sequences were concatenated and Mega5 [67] software is used to perform the phylogeny (alignment by Muscle algorithm, manual removal of non-conserved positions, tree building with NJ and ML methods with 150 bootstraps, comparison of phylogenies and selection of a consensus (Additional file 3)).

Thanks to information about compartmentalization in bacteria, we reconstructed the ancestral state of cell plan with parsimony method (Mesquite software [68] and Phytools package on R software [69]. Phytools allowed the reconstruction of ancestral sates of discrete character by a method of Maximum likelihood). The tree obtained allowed us to date the different events occurred during evolution of the phyla studied (appearance and disappearance of compartmentalization, conversion to intracellular allopatric or sympatric lifestyle).

### Mobilome study

We studied 9 elements directly or indirectly implicated in the horizontal transfers: phages (complete or incomplete), conjugation genes and plasmids, these three elements are involved in entrance of foreign sequences in bacteria; Transposases, integrases and CRISP (candidate or confirmed), regulating positively or negatively the sequences integration in recipient genome; and tRNA used for sequences translation. Different databases were used as RepBase [70] and CRISPfinder [71] to detect the mobilome elements (the complete list of databases used is presented in the Additional file 4). For each element we counted the quantity in each species; if this quantity is related to the genome size (CRISPs, transposases, integrases and conjugation genes), we calculated the proportion of these elements in the genomes. We used the same statistical tests as those used for HGT analysis, in order to determine the elements overrepresented in the different classes of bacteria.

### HGT detection

OrthoMCL [72] was used for construction of orthologous groups. Genes absent to all orthologous groups are either acquired by HGT or generated *de novo* (ORFans). Blast against nr database allowed the identification genes presenting no identifiable homologous genes (*e*-value < 10-4 and coverage > 50 %), considered as ORFans.

Phylopattern [73] pipeline allowed the detection of genetic events in four steps: comparison between species tree and each orthologous groups; detection of missing species in orthologous groups; reconstruction of ancestral state for each proteins, based on the pattern of presence/absence of sequences in modern species; identification of two types of genetic events: gains and losses. Gains could be HGT, de novo genes, or artifacts. We focused on gene gains and Blast [74] each sequences against nr database (NCBI). HGT candidates were identified thanks to filtering on Blast results: For each blast results, sequences derived from phylum where gene gain was detected were removed (for example, if gain was identified in *Akkermansia muciniphila* (*Verrucomicrobiae*), all sequences in Blast results derived from *Verrucomicrobiae* were removed). Sequences with *e*-value > 10-5, coverage < 60 % or identities < 30 % were also removed. Then, species of the first ten sequences remaining in Blast result were identified. If species identified do not belong to one of the sister phyla studied, gene gain is probably an HGT (for example if gain was identified in *Akkermansia muciniphila*, gain is considered as HGT only if the first ten hits do not belong to the phyla *Planctomycetes* or *Chlamydiae*). The position on gains in species tree allows to differentiate HGT occurred only in modern species (specific HGT) and HGT occurred in the ancestor (non specific HGT). We calculated the quantity of HGT and the percentage of proteins present in each species, due to transfers. For each HGT detected (recent or ancient), we determined with which organism the transfers were realized.

For each elements studied (HGT quantity, proportions, partners and mobilome), we performed a comparison of variances between the different groups of bacteria (based on cell plan). We tested the normality of data distribution by the shapiro-Wilk test and the homogeneity of variance by Levene test. These tests were followed by a comparison of variance between the different classes, in order to evaluate if there was a significant difference between them (Kruskal test for data without a normal distribution and ANOVA test for data with a normal distribution). Tests of Nemenuyi (data not normally distributed) or Tukey (data normally distributed) were performed to obtain a comparison of each pairs of classes. We realized also these statistical analysis with bacterial groups based on the phyla, to determine if classes based on phyla present the same results that classes based on cell plan. If it had been the case, it would have been impossible to determine if differences observed were related to cell plan or to phylogenetic relations.

## Reviewers’ comments

### Reviewer summary

#### Reviewer 1 (Mircea Podar)

“In their study, Pinos et al tested their hypothesis that cellular compartmentalization, observed in some representatives of the PVC super phylum, is correlated to the frequency of horizontal gene transfer. The authors performed ultrastructural analyses of a representative from the Chlamydia and one from Verrucomicrobiae, which were shown to be compartmentalized. That information was combined with results from literature on other PVC species and was correlated with phylogenomic analyses on HGT frequencies, based on completed genomes. The result indicates that compartmentalization is in fact not significantly impacting HGT, which has a stronger correlation with the organismal life-style.

The study is interesting and original, even though the conclusion did not support the original hypothesis. It does provide a foundation for additional tests and focused study of related organisms/genomes, which may increase the signal to link compartmentalization to more subtle evolutionary genomic effects.”

#### Reviewer 2 (Olivier Tenaillon)

“In the manuscript entitled “Compartmentalization in PVC super-phylum: evolution and impact”, Sandrine Pinos and colleagues use both microscopy and a phylogenetic approach to study Compartmentalization and its potential impact on horizontal gene transfer.

The idea is interesting, but the paper is not very clear in its present form. The usage of microscopy is not introduced properly, statistics and methods are over-simplified and impossible to reproduce. I would consider therefore that the paper needs very substantial rewriting and the phylogenetic analysis should include uncertainty in the phylogeny.”

### Reviewer recommendations to authors

**Reviewer 1 (Mircea Podar):** The manuscript needs extensive work in correcting the many language errors (spelling, syntax, grammar). It also does not provide sufficient detail to evaluate the power of the phylogenomic analyses that were performed. While I like the concept of this study and the overall approach, the manuscript requires a complete overhaul.

**Reviewer 2 (Olivier Tenaillon)**: How robust is the tree? Before being able to conclude on the ancestry of a trait, the robustness of the phylogeny has to be tested. In my experience a 16S phylogeny is not very robust and much more loci should be involved in the reconstruction, bootstrap values should be presented. Character reconstruction should also take into account the tree uncertainty. This is critical for both figures and both character mapping procedures.

**Reviewer 2 (Olivier Tenaillon)**: line 219: “If species identified do not belong to one of the close phyla studied, gene gain is probably an HGT” how close is close, the threshold use should be clearly defined?”

Author’s response: *The different comments and questions concerning our phylogenomic strategy indicate an important problem in our presentation of this method and its results. In order to correct these problems, we expanded the methodology part of phylogenomic strategy, by adding indications and some examples (we detailed some points, as what we want to say by “If species identified do not belong to one of the close phyla studied, gene gain is probably an HGT”, in this case, “close phyla” refers to species belonging to the sister phylum). We also added, in the Additional file 3, phylogenies of PVC bacteria and Bacteroidetes-Spirochaetes-Chlorobi, with the indication of bootstrap values. These phylogenies are more robust than those of the first version, due to the use of ten supplementary markers, for alignment and phylogenetic reconstruction. We want to underline that some results obtained by our phylogenomic method are convergent with results already observed in previous studies (line 155–161), this is an indication of the relative efficiency of our method.*

*The improvement of the phylogeny allowed a better reconstruction of cell plan evolution in PVC bacteria. However, In order to take into account the problem of phylogenetic uncertainties, we used another method of ancestral reconstruction that not only considers these uncertainties, but also provides the probabilities of each state of cell plan at each nodes of the tree. The results of this analysis were included in the Figure 2, they led to a better vision of PVC bacteria evolution and highlighted the importance of microscopic data.*

**Reviewer 2 (Olivier Tenaillon)**: Introduction - The link between horizontal gene transfer and compartmentalization is not clearly stated in the introduction. Two arguments are mentioned, the second one a physical limit is somehow simple to understand, but the first one remains enigmatic: “the presence or absence of compartmentalization in the different PVC bacteria and the reconstruction of ancestral states”. What is meant by this sentence is not clear to me. I think developing more on the subject would improve the introduction.

Author’s response: *This sentence is difficult to explain without the knowledge about the compartmentalization and the lifestyle of PVC bacteria, however, it could be long to detail all these information in introduction. Consequently, we decided to remove this sentence from the introduction, we presented and explained it in the results part. This sentence resumed the idea that if Chlamydiae are the only non compartmentalized PVC bacteria sequenced and also the only intracellular bacteria of this super-phylum, if the compartmentalization protects genomes against HGT, its lost in the intracellular species could be related to the isolation of these bacteria that induces a low proportion of HGT, and leads to the uselessness of compartmentalization. So, the bibliographic knowledge about the presence or absence of compartmentalization in species suggests the hypothesis of a role of the ICM in protection against HGT.*

**Reviewer 2 (Olivier Tenaillon):** Results - The result section starts with the description of microscopic structures. It is not introduced in anyway. The two first paragraphs should be inverted: first mention that the compartmentalization is unknown in some species, then present the evidence for the ones lacking information.

Discussion - again no clear explanation of the interest of the microscopy.

Author’s response: *As the result of the comments of reviewers, we decided to reorganize the results of microscopic analyzes to improve our presentation. We insisted on the importance of these data in our study by adding explanations of their use in ancestral reconstruction (71–80) and their role in the study of the relation between HGT and compartmentalization (97–99). We realized a more detailed ancestral state reconstruction, with a more complex method (analysis performed thanks to R software, with phylotools package, by a maximum likelihood method) that provided the probabilities of each cell plan states at each nodes of the tree. These analyzes highlighted the importance to add our data to the study, indeed our supplement of information allowed to improve significantly the probability of compartmentalization for several nodes of tree.*

**Reviewer 2 (Olivier Tenaillon):** Results - The term allopatric and sympatric should clearly be defined in the present context and not in the method. Being an evolutionary biologist rather than a microbiologist, I do not support the use of these terms that refer to speciation and not to accessibility of foreign DNA, but as the author have previously used them… It should nevertheless be explicitly defined, with the present definition it is not clear who has access to other microbes.

Author’s response: *Indeed the terms allopatry and sympatry needed to be clearly defined, we added the complete definition in methodology part and we also put a short definition in the results part.*

**Reviewer 2 (Olivier Tenaillon)**: The mobilome analysis are not described enough to be reproducible.

Author’s response: *We improved the paragraphs dedicated to the mobilome analysis by adding explanations about our strategy (elements selected and calculation of proportions). We completed these explanations by a table of the databases used to retrieve the mobilome elements, available in the Additional file 4.*

**Reviewer 2 (Olivier Tenaillon)**: “line 206 “Genes absent to all orthologous groups are either acquired by HT or generated de novo”: this sentence is not clear. Sometimes HT is used sometimes HGT.

Author’s response: *The term HT indicates an horizontal transfer of sequence, this sequence could contain one or more genes (average of genes implicated by transfer is 1.5). The term HGT refers to a transfer of one gene. As the use of these two terms complicated our manuscript, we decided to use only the term of HGT, and we adapted our data in consequence.*

**Reviewer 2 (Olivier Tenaillon)**: line 206 NR database: the author should say what it is and use either nr or NR.

Author’s response: *Indeed we used the nr database, we corrected this mistakes in manuscript.*

**Reviewer 2 (Olivier Tenaillon)**: Figure - species names should be written in full and in italic with no underscore. Line 70–75 Statistics should be given to compare “sympatric” groups.

Author’s response: *The figures were corrected and we added the statistics for sympatric bacteria comparison in the paragraph.*

## Additional files

Additional file 1: Mobilome distribution in the different bacterial groups according lifestyle and cell plan. The different graphics allow a better sight of the different levels for each mobilome feature. Red stars show significant differences between groups. (PDF 175 kb) Additional file 2: Characteristics of studied species. This table presents the lifestyles, genomes and proteomes features of each species studied. (PDF 125 kb) Additional file 3: Phylogeny of PVC bacteria and phylogeny of *Bacteroidetes-Chlorobi-Spirochaetes*. The phylogenies of studied bacteria were realized with Maximum likelihood method, thanks to Mega6 software, the bootstraps values are indicated at each node. (PDF 234 kb) Additional file 4: Databases used for the mobilome study. The mobilome elements studied are indicated in the first column, the second column contains the corresponding database and the third column presents the url of websites for these databases. (PDF 177 kb)
