# Supplementary figures and images for: Compartmentalization in PVC super-phylum: evolution and impact
Source: Biol Direct. 2016 Aug 9;11:38. doi: 10.1186/s13062-016-0144-3 (PMC4977879; doi:10.1186/s13062-016-0144-3)

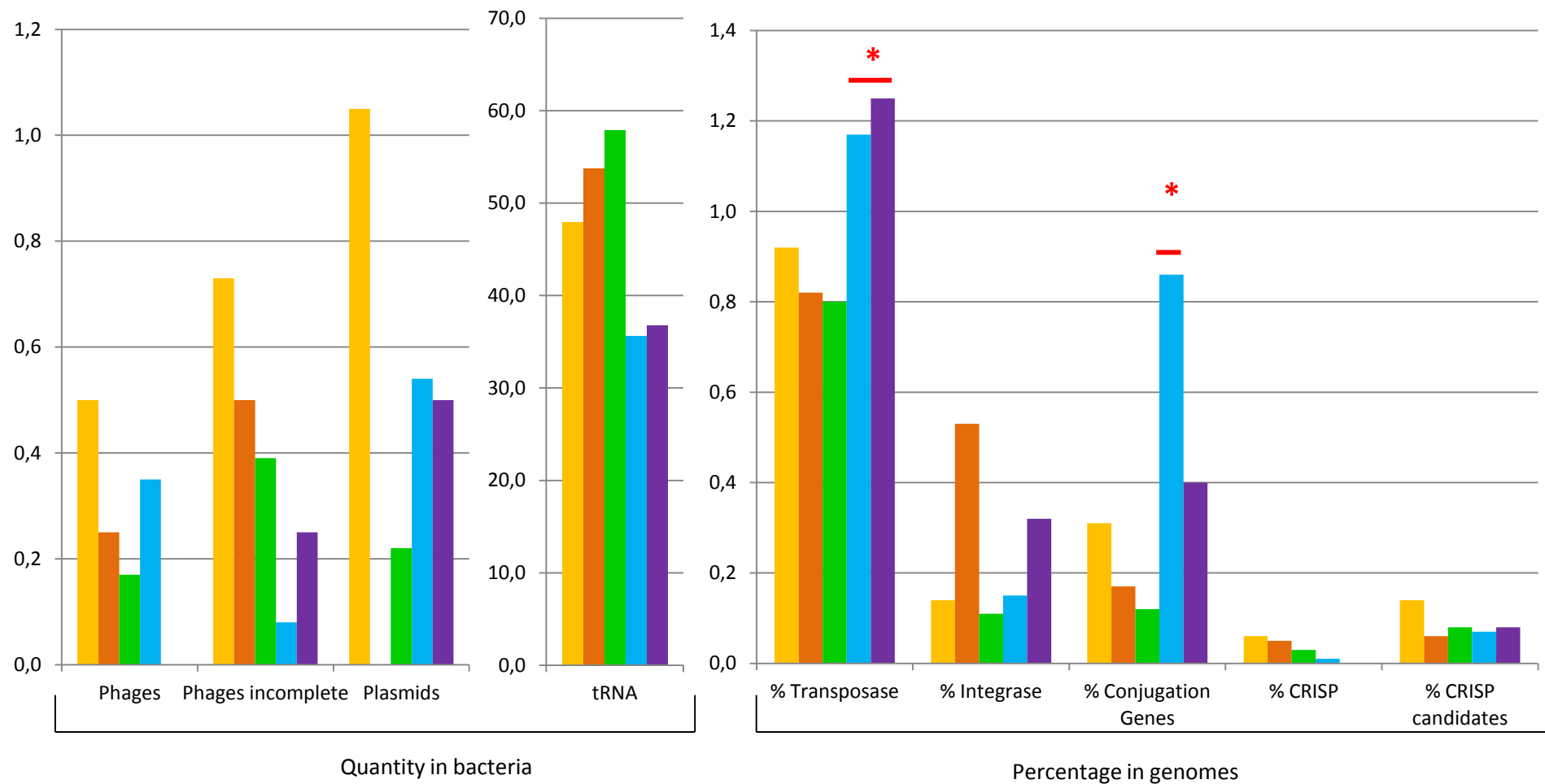

Supplement: Additional file 1: — Mobilome distribution in the different bacterial groups according lifestyle and cell plan. The different graphics allow a better sight of the different levels for each mobilome feature. Red stars show significant differences between groups. (PDF 175 kb) [file 13062_2016_144_MOESM1_ESM.pdf]

## Phylogeny of PVC bacteria

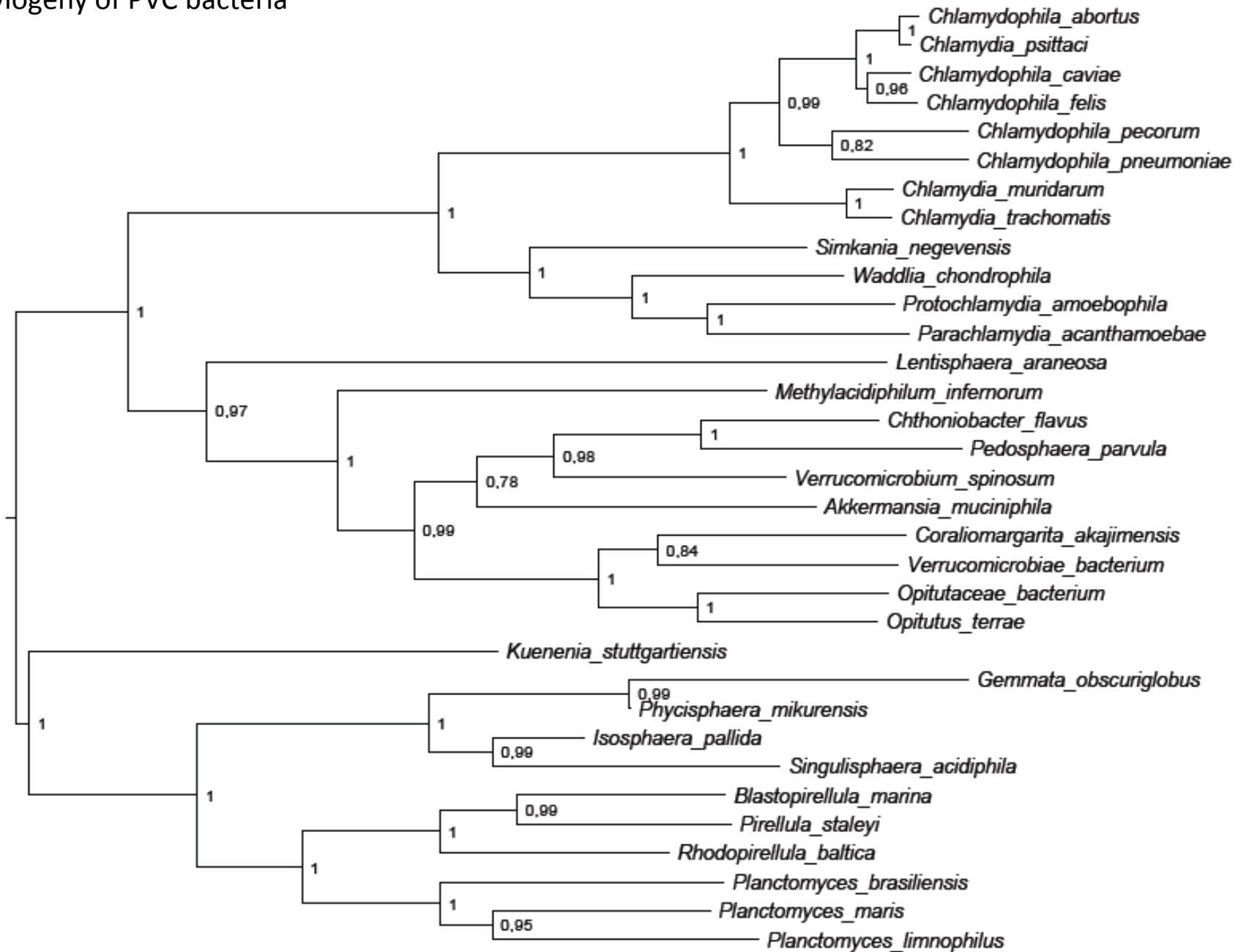

# Phylogeny of *Bacteroidetes*, *Spirochaetes* and *Chlorobi*

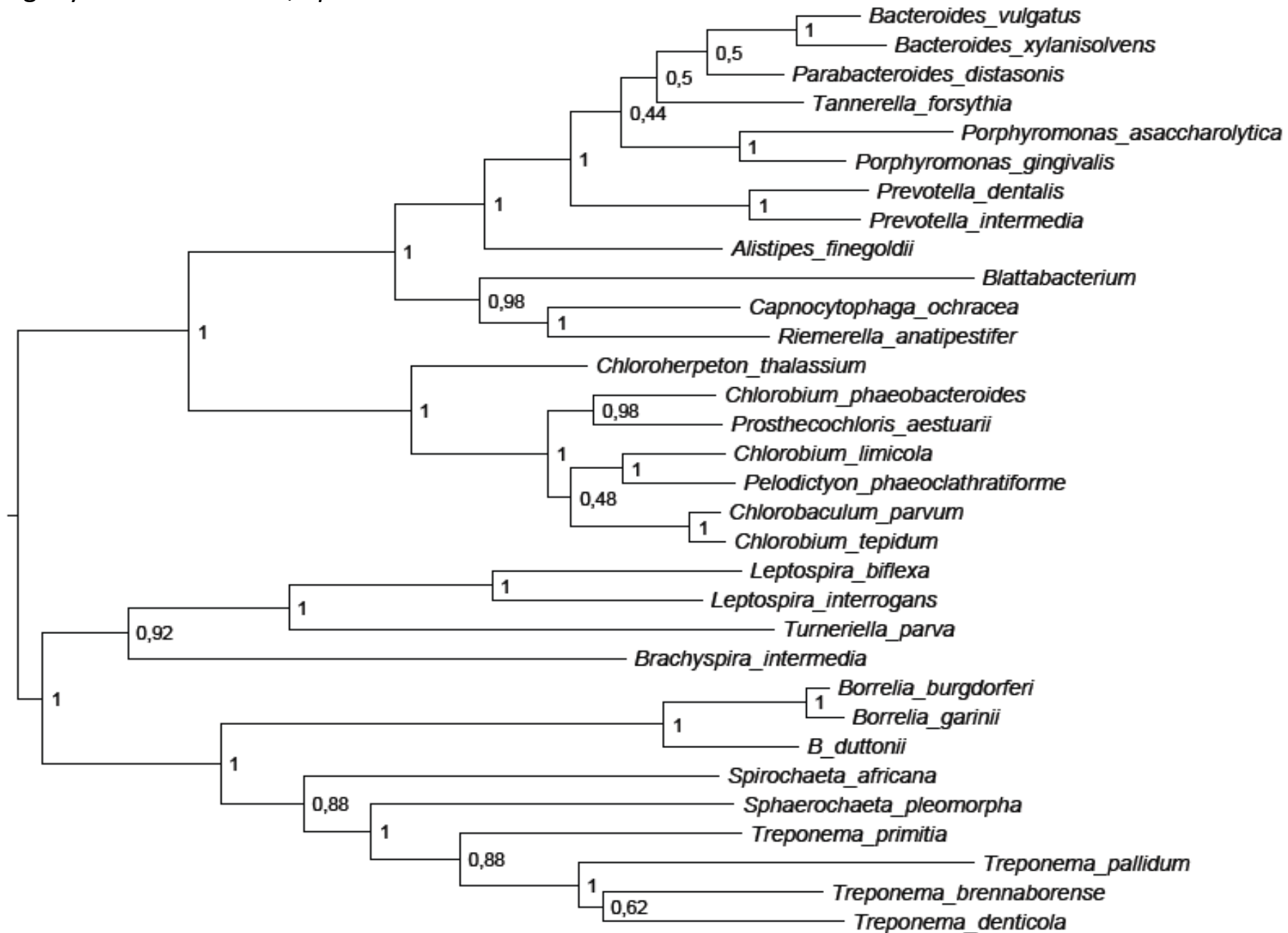

Supplement: Additional file 3: — Phylogeny of PVC bacteria and phylogeny of Bacteroidetes-Chlorobi-Spirochaetes. The phylogenies of studied bacteria were realized with Maximum likelihood method, thanks to Mega6 software, the bootstraps values are indicated at each node. (PDF 234 kb) [file 13062_2016_144_MOESM3_ESM.pdf]
