# Supplementary material for: Compartmentalization in PVC super-phylum: evolution and impact
Source: Biol Direct. 2016 Aug 9;11:38. doi: 10.1186/s13062-016-0144-3 (PMC4977879; doi:10.1186/s13062-016-0144-3)
Supplement: Additional file 2: — Characteristics of studied species. This table presents the lifestyles, genomes and proteomes features of each species studied. (PDF 125 kb) [file 13062_2016_144_MOESM2_ESM.pdf]

Species	Lifestyles	Genome identfifiers	Genome size	Proteome sizes
Candidatus Protochlamydia amoebophila	Intracellular	NC_005861	2.4	2031
Parachlamydia acanthamoebae UV-7	Intracellular	NC_015702	3.1	2782
Simkania negevensis Z	Intracellular	NC_015713	2.6	2512
Waddlia chondrophila WSU 86-1044	Intracellular	NC_014225	2.1	1956
Opitutaceae bacterium TAV1	Extracellular	NZ_AHKS00000000	7.1	5830
Treponema primitia ZAS-2	Extracellular	NC_015578	4.1	3523
Verrucomicrobium spinosum DSM 4136	Extracellular	NZ_ABIZ00000000	8.2	6477
Blattabacterium sp (Blaberus giganteus)	Intracellular	NC_017924	0.6	576
Chthoniobacter flavus Ellin428	Extracellular	NZ_ABVL00000000	7.9	6716
Opitutus terrae PB90-1	Extracellular	NC_010571	6.0	4612
Pedosphaera parvula Ellin514	Extracellular	NZ_ABOX00000000	7.4	6510
Gemmata obscuriglobus UQM 2246	Extracellular	NZ_ABGO00000000	9.2	7969
Isosphaera pallida ATCC 43644	Extracellular	NC_014962	5.5	3722
Methylacidiphilum infernorum V4	Extracellular	NC_010794	2.3	2472
Verrucomicrobiae bacterium DG1235	Extracellular	NZ_ABSI00000000	5.8	4909
Leptospira biflexa serovar Patoc	Extracellular	NC_010842 NC_010845	4.0	3600
Leptospira interrogans serovar Lai str. 56601	Extracellular	NC_004342 NC_004343	4.7	3702
Turneriella parva DSM 21527	Extracellular	NC_018020.1	4.4	4139
Alistipes finegoldii DSM 17242	Extracellular	NC_018011	3.7	3110
Akkermansia muciniphila ATCC BAA-835	Extracellular	NC_010655	2.7	2138
Borrelia burgdorferi JD1	Extracellular	NC_017403	1.5	1458
Borrelia garinii BgVir	Extracellular	NC_017717	1.0	950
Brachyspira intermedia PWS/A	Extracellular	NC_017243	3.3	2872
Bacteroides vulgatus ATCC 8482	Extracellular	NC_009614	5.2	4066
Bacteroides xylanisolvens XB1A	Extracellular	NC_021017	6.0	4405
Capnocytophaga ochracea DSM 7271	Extracellular	NC_013162	2.6	2171
Porphyromonas asaccharolytica DSM 20707	Extracellular	NC_015501	2.2	1699
Prevotella dentalis DSM 3688	Extracellular	NC_019960 NC_019968	3.4	2544
Parabacteroides distasonis ATCC 8503	Extracellular	NC_009615	4.8	3849
Porphyromonas gingivalis W83	Extracellular	NC_002950	2.3	1909
Prevotella intermedia 17	Extracellular	NC_017860 NC_017861	2.7	2266
Riemerella anatipestifer ATCC 11845 = DSM 15868	Extracellular	NC_014738	2.2	1972
Treponema brennaborense DSM 12168	Extracellular	NC_015500	3.1	2531
Treponema denticola ATCC 35405	Extracellular	NC_002967	2.8	2767
Tannerella forsythia ATCC 43037	Extracellular	NC_016610	3.4	3001
Treponema pallidum subsp. pallidum DAL-1	Extracellular	NC_016844	1.1	1056
Chlamydophila abortus S26/3	Intracellular	NC_004552	1.1	932
Chlamydophila caviae GPIC	Intracellular	NC_003361	1.2	1005
Chlamydophila felis Fe/C-56	Intracellular	NC_007899	1.2	1013
Chlamydia muridarum Nigg	Intracellular	NC_002620	1.1	911
Chlamydophila pecorum E58	Intracellular	NC_015408	1.1	988
Chlamydophila pneumoniae J138	Intracellular	NC_002491	1.2	1069
Chlamydia psittaci NJ1	Intracellular	NC_018626	1.2	1052
Chlamydia trachomatis 434/Bu	Intracellular	NC_010287	1.0	885
Blastopirellula marina DSM 3645	Extracellular	NZ_AANZ00000000	6.7	6025
Coraliomargarita akajimensis DSM 45221	Extracellular	NC_014008	3.8	3120
Chlorobium limicola DSM 245	Extracellular	NC_010803	2.8	2434
Chlorobaculum parvum NCIB 8327	Extracellular	NC_011027	2.3	2043
Chlorobium phaeobacteroides BS1	Extracellular	NC_010831	2.7	2469
Chlorobium tepidum TLS	Extracellular	NC_002932	2.2	2245
Chloroherpeton thalassium ATCC 35110	Extracellular	NC_011026	3.3	2710
Candidatus Kuenenia stuttgartiensis	Extracellular	_	4.2	4663
Lentisphaera araneosa HTCC2155	Extracellular	NZ_ABCK00000000	6.0	5104
Prosthecochloris aestuarii DSM 271	Extracellular	NC_011059	2.6	2327
Planctomyces brasiliensis DSM 5305	Extracellular	NC_015174	6.0	4750
Planctomyces limnophilus DSM 3776	Extracellular	NC_014148	5.5	4258
Planctomyces maris DSM 8797	Extracellular	NZ_ABCE00000000	7.8	6480
Phycisphaera mikurensis NBRC 102666	Extracellular	NC_017080	3.9	3282
Pelodictyon phaeoclathratiforme BU-1	Extracellular	NC_011060	3.0	2707
Pirellula staleyi DSM 6068	Extracellular	NC_013720	6.2	4717
Rhodopirellula baltica SH 1	Extracellular	NC_005027	7.2	7325
Singulisphaera acidiphila DSM 18658	Extracellular	NC_019892	9.8	7251
Spirochaeta africana DSM 8902	Extracellular	NC_017098	3.3	2782
Sphaerochaeta pleomorpha str. Grapes	Extracellular	NC_016633	3.6	3159
