# Supplementary material for: Compartmentalization in PVC super-phylum: evolution and impact
Source: Biol Direct. 2016 Aug 9;11:38. doi: 10.1186/s13062-016-0144-3 (PMC4977879; doi:10.1186/s13062-016-0144-3)
Supplement: Additional file 4: — Databases used for the mobilome study. The mobilome elements studied are indicated in the first column, the second column contains the corresponding database and the third column presents the url of websites for these databases. (PDF 177 kb) [file 13062_2016_144_MOESM4_ESM.pdf]

Mobilomes element	Databases	Website URL
Plasmids	GOLD,<br>NCBI	https://gold.jgi.doe.gov/<br>http://www.ncbi.nlm.nih.gov/genome/
Phages	PHAST<br>PhageFinder	http://phast.wishartlab.com/<br>http://phage-finder.sourceforge.net/
Conjugation genes	SecReT4	http://db-mml.sjtu.edu.cn/SecReT4/index.php
Transposases	NCBI,<br>RepeatFinder	http://www.ncbi.nlm.nih.gov/protein<br>http://www.cbcb.umd.edu/software/RepeatFinder/
Integrases	NCBI<br>ReapetFinder	http://www.ncbi.nlm.nih.gov/protein<br>http://www.cbcb.umd.edu/software/RepeatFinder/
CRISP	CRISPFinder	http://crispr.i2bc.paris-saclay.fr/Server/
tRNA	GtRNAdb<br>tRNAscan-SE	http://gtrnadb.ucsc.edu/<br>http://lowelab.ucsc.edu/tRNAscan-SE/
